# Clinical and Immunological Profiles With Treatment Patterns in Systemic Lupus Erythematosus Patients in the Eastern Part of India: A Retrospective, Hospital-Based, Cross-Sectional Study

**DOI:** 10.7759/cureus.103093

**Published:** 2026-02-06

**Authors:** Swetalina Pradhan, Debopriya Paul, Rakhee Gupta, Suvesh Singh, Rashid Shahid

**Affiliations:** 1 Department of Dermatology, All India Institute of Medical Sciences, Patna, Patna, IND; 2 Department of Dermatology, All India Institute of Medical Sciences, Patna, Bihar, IND

**Keywords:** le, lupus nephritis, rituximab, sle, systemic lupus erythematosus

## Abstract

Background: Systemic lupus erythematosus (SLE) is a multisystem autoimmune disorder with a wide range of clinical and immunological abnormalities. Several studies have been conducted on the clinical and immunological profile of SLE patients from different parts of India; however, similar studies are lacking from the Eastern region of the country.

Aims and objectives: The primary objective was to study the clinical manifestations of SLE patients and to determine their immunological profile. The secondary objective was to find an association between clinical features and immunological profile.

Materials and methods: A retrospective cross-sectional study was conducted at a tertiary care hospital in eastern India, analyzing all data from January 2019 to September 2023, where the diagnosed cases based on Systemic Lupus International Collaborating Clinics (SLICC) criteria were included. Records of 79 patients were evaluated, and their demographic profile, clinical manifestations, biochemical parameters, immunological parameters (antinuclear antibodies (ANA) screening and ANA profile) and treatment details were analysed. Data analysis was performed using IBM SPSS Statistics for Windows, version 20.0 (IBM Corp., Armonk, NY, USA).

Results: Out of 79 patients, there was a female preponderance (6.9:1). The mean age was 29.5 ± 12.8 years. Common clinical manifestations included diffuse non-scarring alopecia 68 (86.1%), joint pain 67 (84.8%), fever 63 (79.7%), and oral ulcers 62 (78.5%). Lupus nephritis (LN) was found in 41 (51.9%) of patients. Anti-nuclear antibodies (ANA) were tested positive among 70 (88.6%) patients. The most commonly detected antibodies were anti-dsDNA 55 (69.6%), anti-U1snRNP 47 (59.5%), anti-Sm 35 (44.3%), anti-Ro 60 35 (44.3%), and anti-Ro52 31 (39.2%). Anti-Sm antibody significantly correlated with lupus nephritis (p=0.002), and anti-dsDNA was associated with maculopapular rash (p=0.012). Our study highlighted the role of rituximab in managing the various disease spectrum of SLE. Conclusion: This study compares the clinical and immunological profiles of patients from eastern India with those of similar other studies. While analysing, we also found a significant association between anti-dsDNA with maculopapular rash (p-value = 0.012) and anti-Sm antibody (25,60.97%) was the most common antibody among LN patients, with a significant association (p-value = 0.002). Our study also highlighted the management of a few challenging cases, like that of hemophagocytic lymphohistiocytosis (HLH), antiphospholipid antibodies (APLA), and central nervous system (CNS) vasculitis.

## Introduction

Systemic lupus erythematosus (SLE) is a multisystem autoimmune disorder with a wide range of clinical and immunological abnormalities [1]. The reported point prevalence of SLE in India is 3.2 per 100,000 people [2]. Skin is one of the most common organs involved in SLE patients [3]. The cutaneous findings are further classified into LE-specific and LE-non-specific according to Gilliam and Sontheimer's histopathologic classification [4]. The LE-specific changes include malar rash, maculopapular rash, photosensitive lupus, toxic epidermal necrolysis variant of LE, discoid lupus, lupus panniculitis, and chilblain lupus, among others [5]. The common LE non-specific lesions include non-scarring diffuse alopecia, oro-nasal ulcers, and Raynaud’s phenomenon [6]. These heterogeneous clinical manifestations of SLE often arise due to a complex interplay of genetic, environmental and hormonal factors [7]. Apart from skin, musculoskeletal, renal, hematological, central nervous system (CNS), and serosal membrane involvement are commonly seen [8]. Lupus nephritis (LN) is observed in 38.3% to 68.9% of SLE patients and is one of the most common factors that determines the morbidity and prognosis of the disease [9]. Being an autoimmune disease, several anti-nuclear antibodies are commonly detected in SLE, which include anti-dsDNA, anti-Smith (anti-Sm), anti-Ro/SSA, anti-La, anti-histone, and anti-ribonucleoprotein (anti-RNP) [1,10]. Treatment options include various immunosuppressive agents and biological agents [11]. Recently, many biological agents, like belimumab and rituximab, have also been approved [12].

Several studies have been conducted on the clinical and immunological profiles of SLE patients from different parts of India; however, reports documenting the same are lacking from Eastern India. In this study, we also determined a significant association between clinical features and immunological profile. The rationale is to predict the development of various clinical manifestations beforehand just by determining the immunological profile.

## Materials and methods

A retrospective analysis of 79 patients with SLE, diagnosed based on Systemic Lupus International Collaborating Clinics (SLICC) criteria, was performed at a tertiary care hospital in eastern India from January 2019 to September 2023. Informed consent was obtained from all adult patients or parents in the case of children. Ethical approval was obtained from the Institute Ethics Committee. The patients were included irrespective of age, sex, and pregnancy status. Patients with overlap of SLE and any other connective tissue disease were excluded from the study. Detailed data of demography, history, clinical profile, biochemical parameters, immunological parameters (antinuclear antibodies (ANA) screening and ANA profile), and treatment details were collected from patient files and pre-filled proforma. ANAs were detected by indirect immunofluorescence assay (IFA) using the ANA HEp-2 kit. A serum dilution of 1:100 was considered the cut-off for this assay. Standard criteria, such as Revised Sapporo (Sydney) criteria used for diagnosis of antiphospholipid antibody syndrome (APS), H-score for hemophagocytic lymphohistiocytosis (HLH), American College of Rheumatology (ACR) criteria for CNS lupus, and lupus hepatitis, were referred for diagnosis in this study.

Statistical analysis: Data entry was done using the Microsoft Excel sheet, separate for each of the inclusive study subjects, and analyzed using IBM SPSS Statistics for Windows, version 20.0 (IBM Corp., Armonk, NY, USA). The qualitative data was summarized in the form of proportions, percentages and ratios. The quantitative data is presented in the form of mean +/- standard deviation or median. Statistical association between clinical features and antibodies was tested using the Chi-square test. A p-value <0.05 was considered to be statistically significant.

## Results

Demographic profile

The majority of cases were seen in the age group 18-40 years (55, 69.6%), followed by 40-60 years (11, 13.9%). Ten patients (12.7%) belonged to the pediatric age group. The mean age of the inclusive population was 29.5 ± 12.8 years with age ranging from 10-75 years. Females (69, 87.3%) outnumbered males with a female-to-male ratio of 6.9:1. The mean duration of the disease was 26.3 ± 27.07 months. The maximum disease duration noted was 144 months (12 years).

Clinical profile

Muco-cutaneous manifestations: Diffuse non-scarring alopecia (68, 86.1%) followed by oral ulcer (62,78.5%) (Figure 2) were the most common muco-cutaneous manifestations.Features of acute cutaneous LE were present in 65 patients (82.3%), of which malar rash (Figure 3) was seen in 55 (69.6%), maculopapular rash in 44 (55.7%), and toxic epidermal necrolysis (TEN) like LE (Figure 4) among three (3.79%) and bullous lesions (Figure 5) in five patients (6.32%).

**Figure 1:**
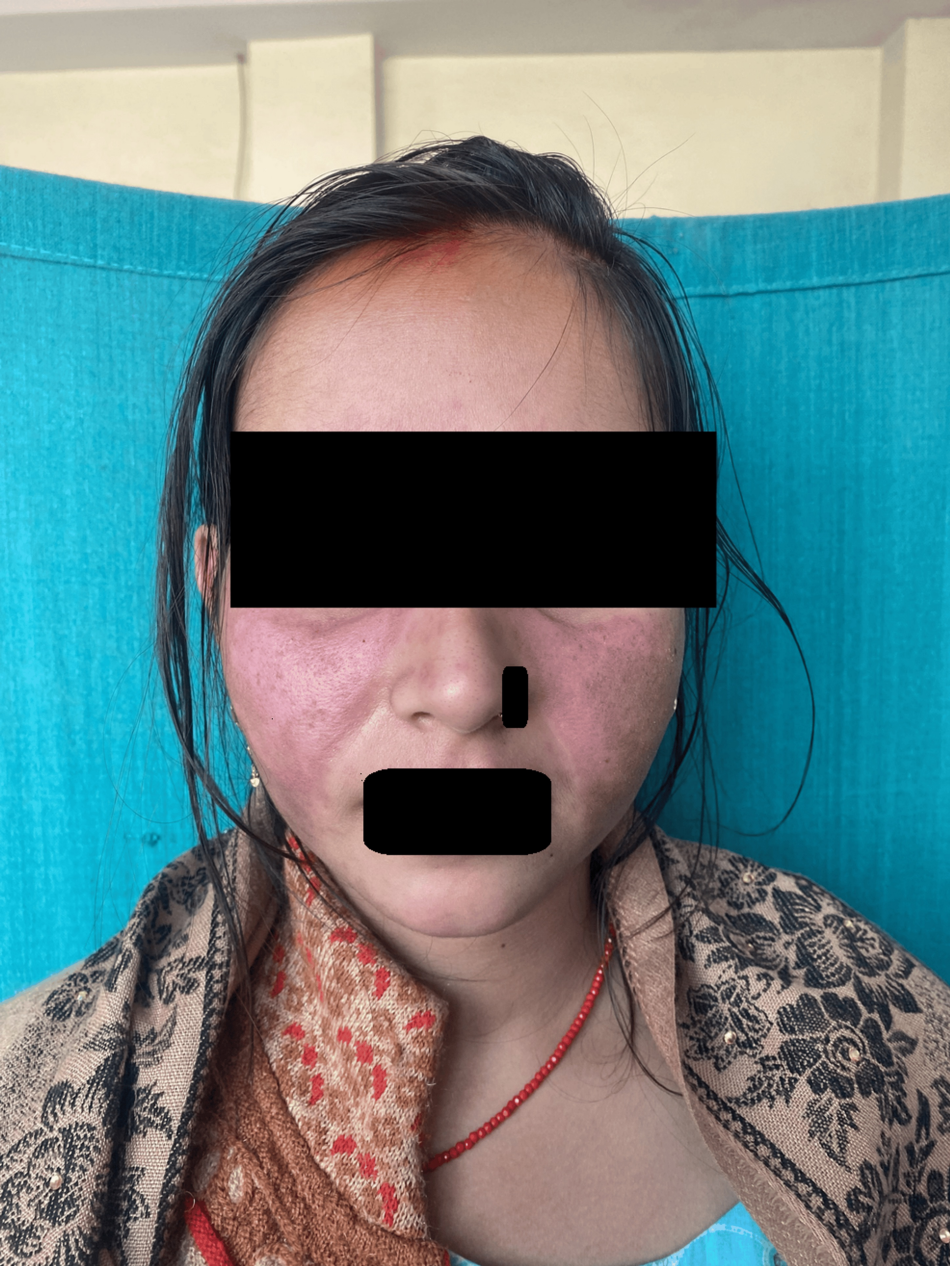
A patient with diffuse well-defined fixed erythema on the face Diffuse well-defined fixed erythema involving the malar region in a symmetrical distribution, with sparing of the nasolabial folds, suggestive of a malar rash. The characteristic sparing of the nasolabial folds, classical of systemic lupus erythematosus, is clearly demonstrated in this image.

**Figure 2:**
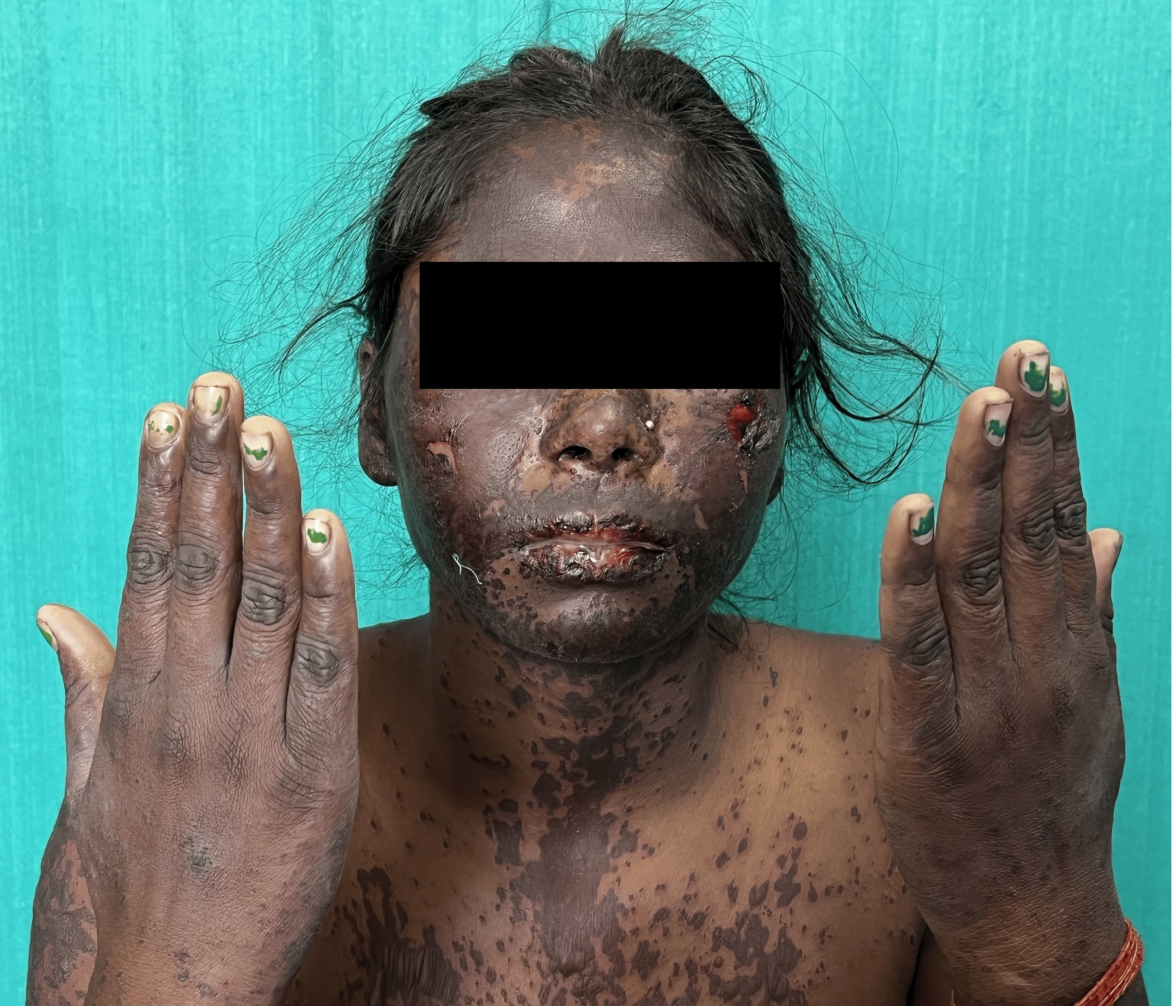
A patient with dusky erythema Dusky erythema involving the face, neck, upper trunk, and extremities, with peeling of skin over the bilateral cheeks, giving rise to erosions.

**Figure 3:**
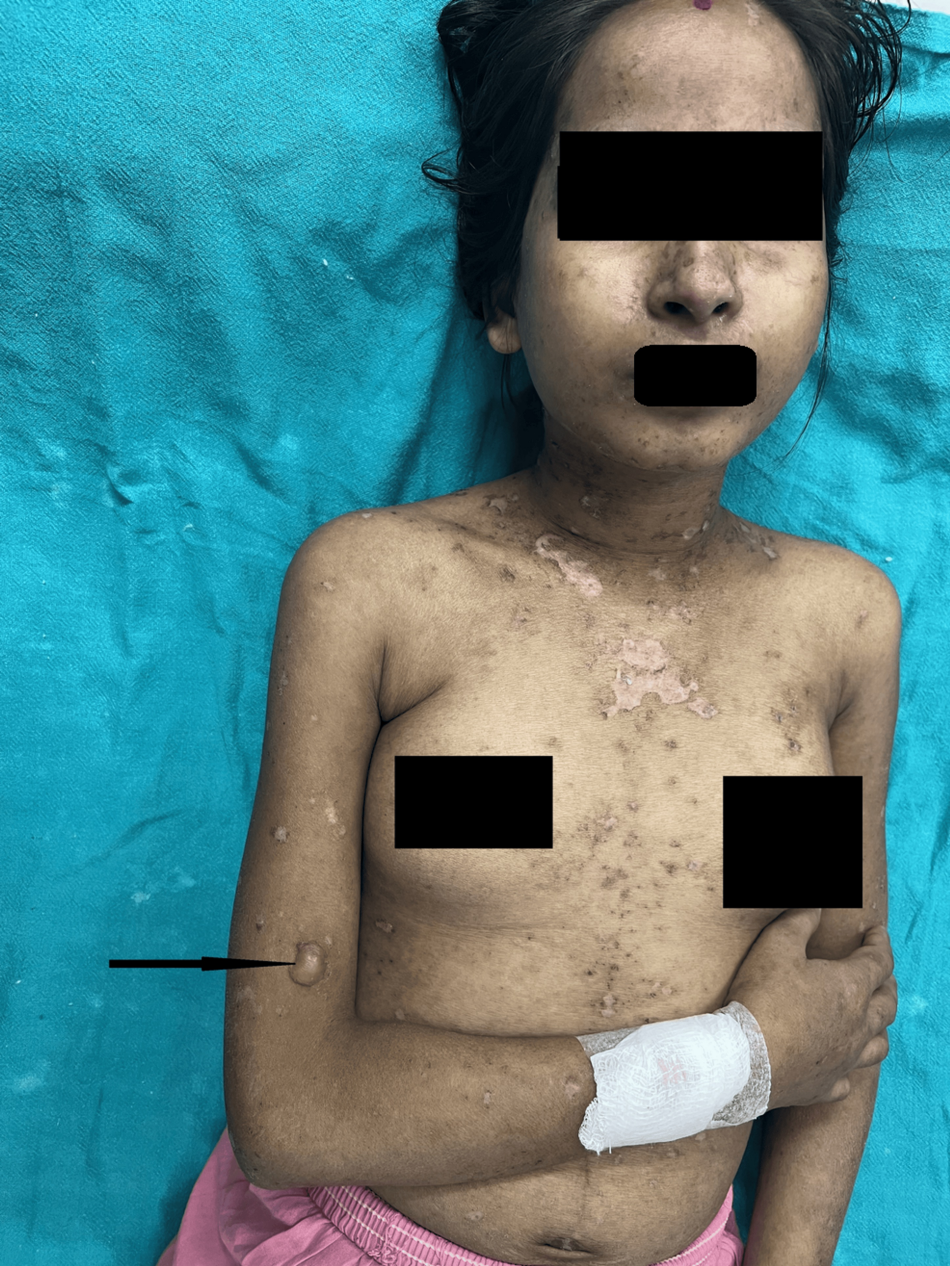
A patient with bullous erosion Single tense bulla present over the right arm with multiple hypopigmented macules with scaling suggestive of healed erosion.

Chronic cutaneous lupus features were seen in 14 patients (17.72%), of which 10 (12.7%) had discoid lupus, three (3.79%) had lupus profundus, and one (1.26%) had lupus panniculitis. Photosensitivity was reported in 54 (68.4%) patients. Palms and soles involvement was documented in 33 (41.7%) patients (Figure 6).

**Figure 4:**
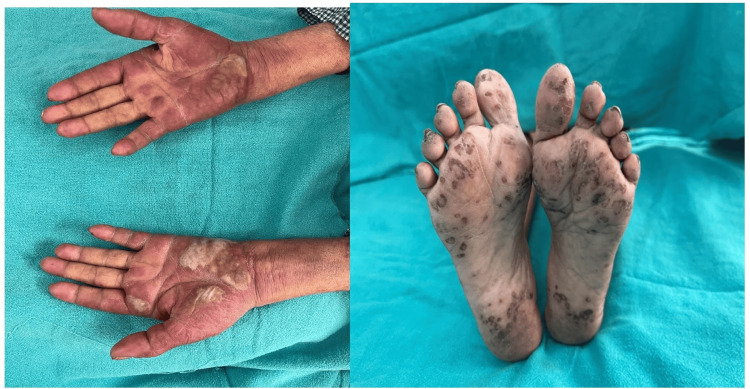
Lupus in palms and soles Left-hand side image: Diffuse erythema involving both palms in a symmetrical pattern with few collapsed bullae and multiple discrete dusky-colored macules present over bilateral soles (right-hand side image).

Mainly three types of lesions encountered in palms and soles: diffuse erythema (18, 54.5%), vasculitic lesions (9, 27.2%), and bullous lesions (6, 18.1%). Nail involvement was found in 46 (58.2%) patients in the form of periungual erythema, onychomadesis, loss of cuticle, transverse ridges, and onychodystrophy. Other less frequent cutaneous findings were vasculitic ulcers (8, 10.1%), urticarial vasculitis (3, 3.79%) (Figure 7), Raynaud’s phenomenon (10, 12.65%), and livedo reticularis (3,3.79%). Massive vulval swelling (Figure 8) was noted in there (3.79%) patients. Two of them had nephrotic range proteinuria, and these patients responded well to a dexamethasone pulse.

**Figure 5:**
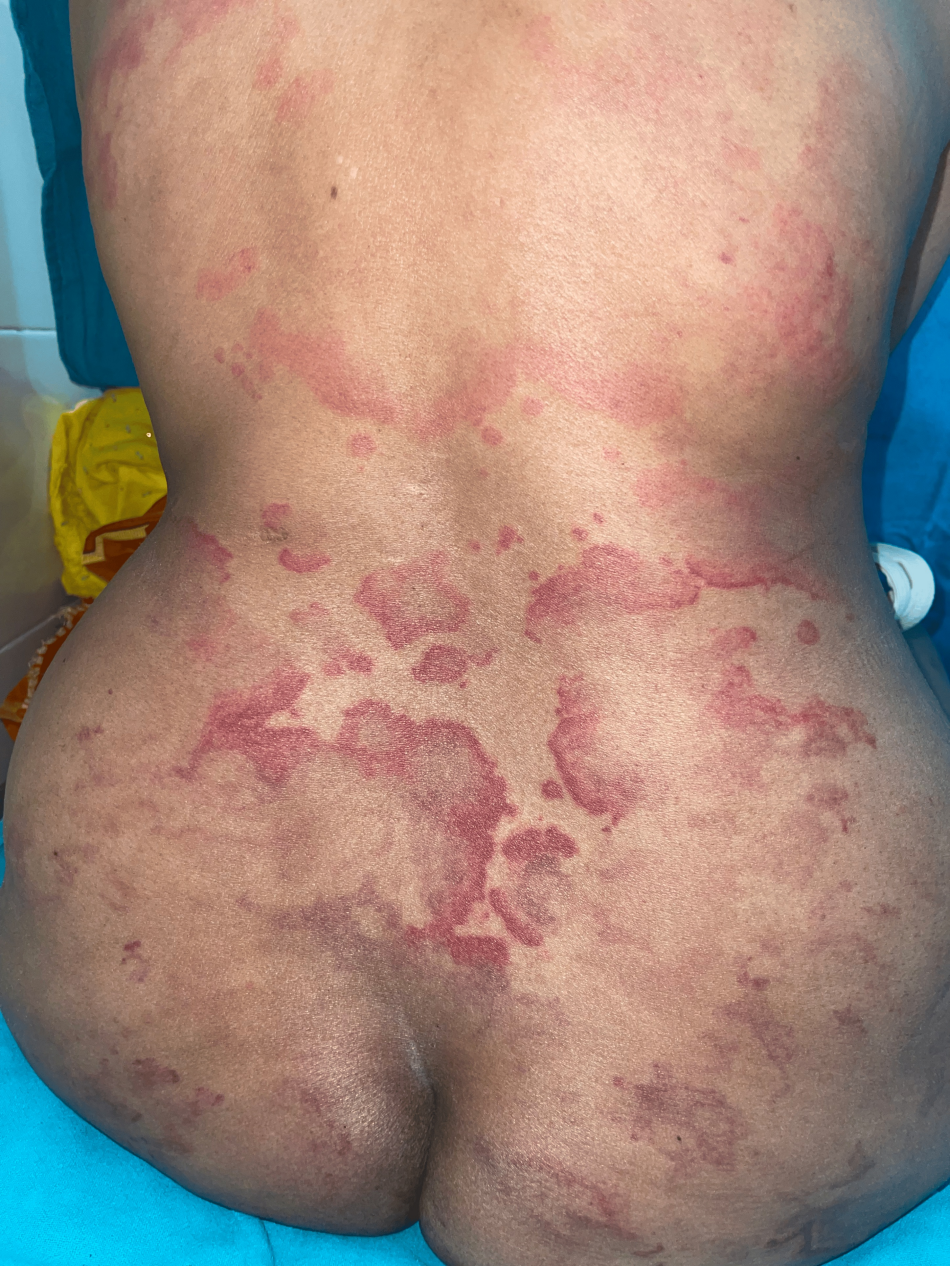
A patient with vasculitic ulcers Multiple discrete to coalescent erythematous plaques with ill- to well-defined margins present over the back and buttocks, which are non-blanchable, suggestive of urticarial vasculitis.

**Figure 6:**
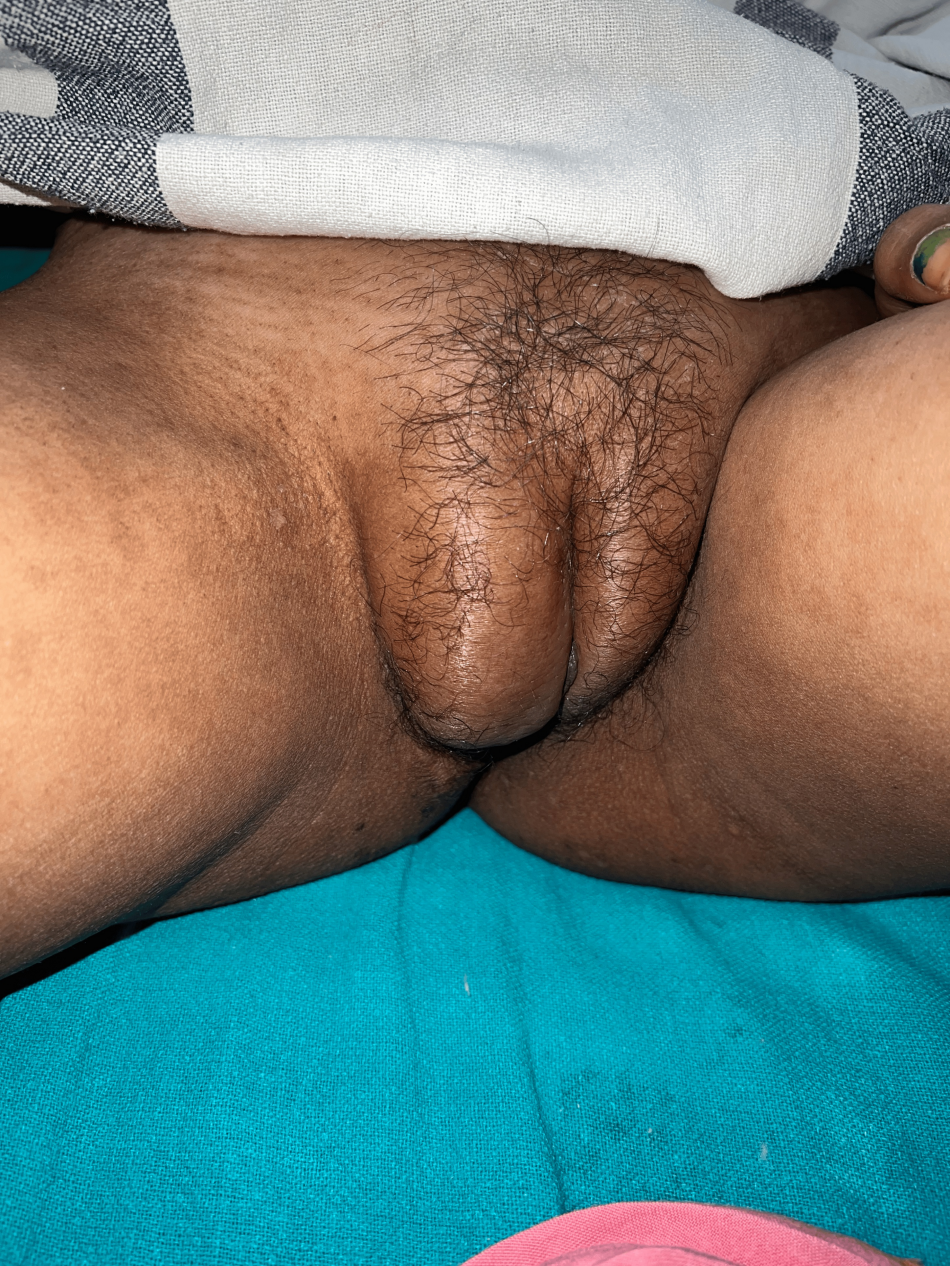
A patient of SLE presenting with vulval edema SLE: Systemic lupus erythematosus.

Systemic manifestations

Joint pain (67, 84.8%) followed by fever (63, 79.7%) were the common systemic symptoms. LN and CNS involvement were detected among 41 (51.9%) and eight (10.1%) patients, respectively. Pleuritis, ascites, and pericarditis were present in three (3.8%), three (3.8%), and two (2.5%) patients, respectively. Figure 9 shows the percentage of patients with different systemic involvement.

**Figure 7:**
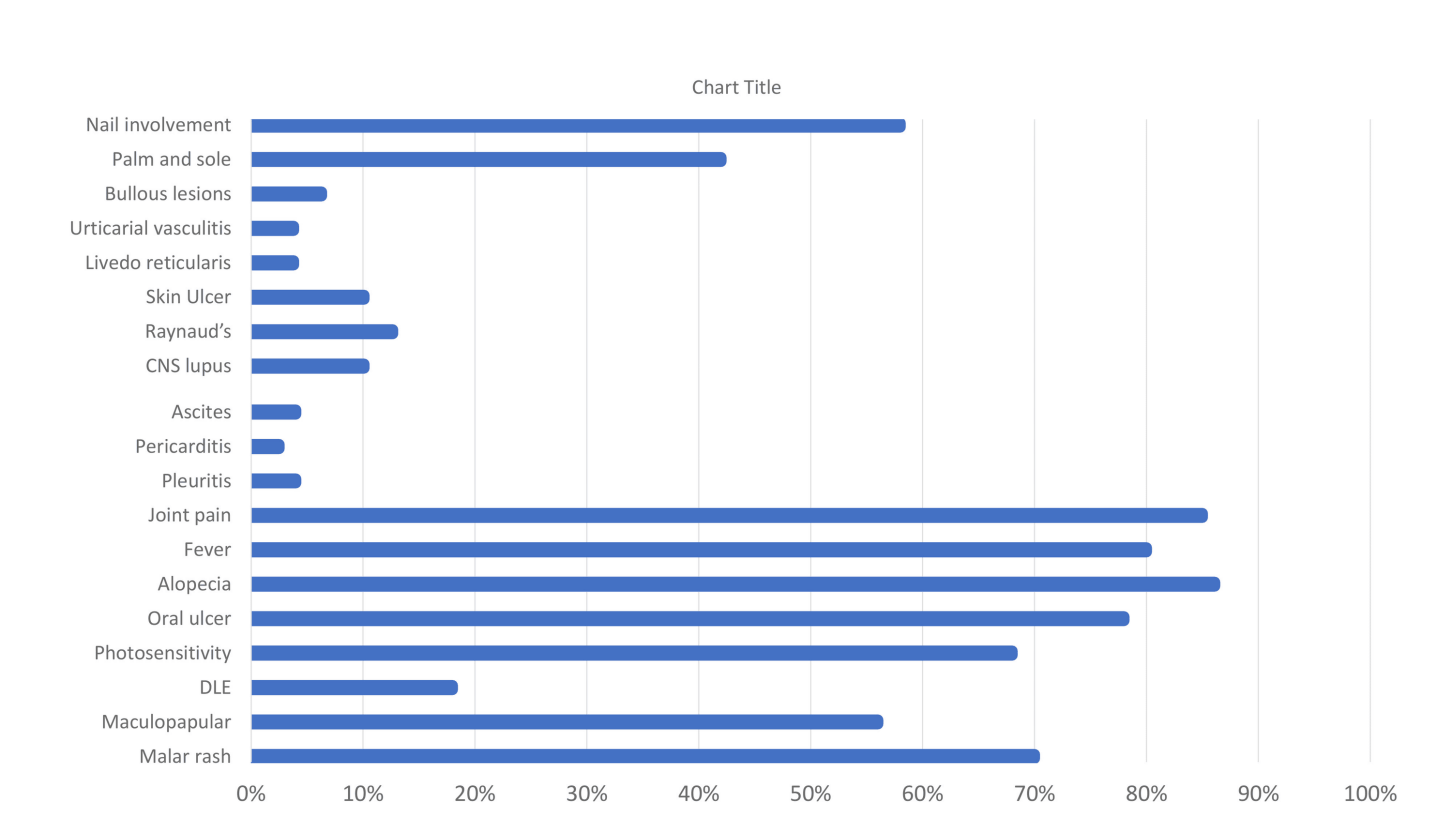
The percentage (%) of patients with different systemic involvement The data has been represented as a percentage (%).

Adult male v/s adult female v/s pediatric population

In adult female patients, the most common mucocutaneous presentations were alopecia (62, 89.9%), malar rash (53, 76.8%), and oral ulcer (51, 73.9%), while among males, oral ulcer (8, 80%) was the most common presentation followed by malar rash (7, 70%) and palms and soles involvement (6, 60%). In children, alopecia (9, 90%) and oral ulcer (8, 80%) were the common clinical findings.

Laboratory parameters

Out of a total of 79 patients, anemia, leukopenia, and thrombocytopenia were present among 62 (78.4%), 15 (18.9%), and 11 (13.9%) patients, respectively. The direct Coombs test was found to be positive in only seven (8.8%) patients. Proteinuria (>500 mg/24 hr urinary protein) was detected among 41 (51.9%) patients, and nephrotic range proteinuria (>3.5 gm/24hr urinary protein) was present among 10 (12.7%) patients. Erythrocyte sedimentation rate (ESR) and C-reactive protein (CRP) were elevated among 57 (72.2%) and 46 (58.2%) patients, respectively. Transaminitis was detected in 41 cases (51.9%) with raised serum glutamic-oxaloacetic transaminase (SGOT) (49.36%) and serum glutamic pyruvic transaminase (SGPT) (36.7%) levels, respectively.

Serious complications

There were two (2.53%) cases of HLH with elevated H-score, which is a known rare complication of SLE. Also, we had three (3.79%) patients of APS, of which two had a previous thrombotic episode.

Radiological findings

Two patients (2.53%) had features of CNS vasculitis in magnetic resonance angiography (MRA) (Figure 10). Four patients (5.06%) had pericardial effusion (in echocardiography), and seven patients (8.86%) had ascites on ultrasound. Pleural effusion was noted in three patients (3.79%) in chest X-ray.

**Figure 8:**
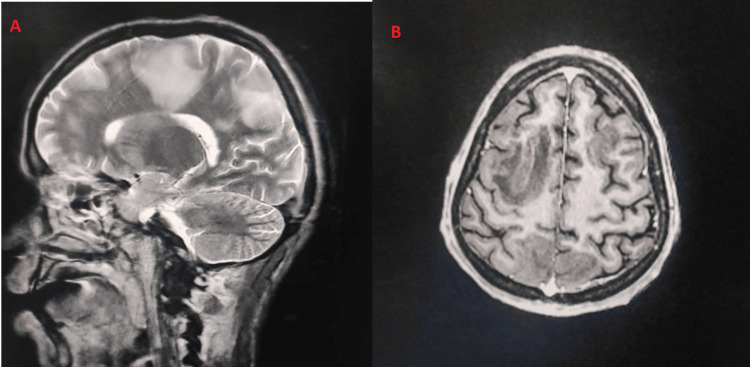
MRA images in a case of CNS vasculitis A) T2-weighted sagittal image of the brain showing patchy areas of hyperintensities involving the right frontal and parietal lobes with predominant involvement of subcortical and deep white matter. Features are consistent with the area of demyelination. B) T1-weighted axial image of the brain shows sharply defined patchy hypointense areas involving the bilateral frontal lobes and parietal lobes. MRA: Magnetic resonance angiography.

Immunological profile

ANA Hep-2 screening was found to be positive in 70 (88.9%) patients. Speckled pattern (49, 62.02%) was the most common, followed by homogenous (29, 36.7%), cytoplasmic (19, 24.1%), and nucleolar (6, 7.6%) patterns (Figure 11).

**Figure 9:**
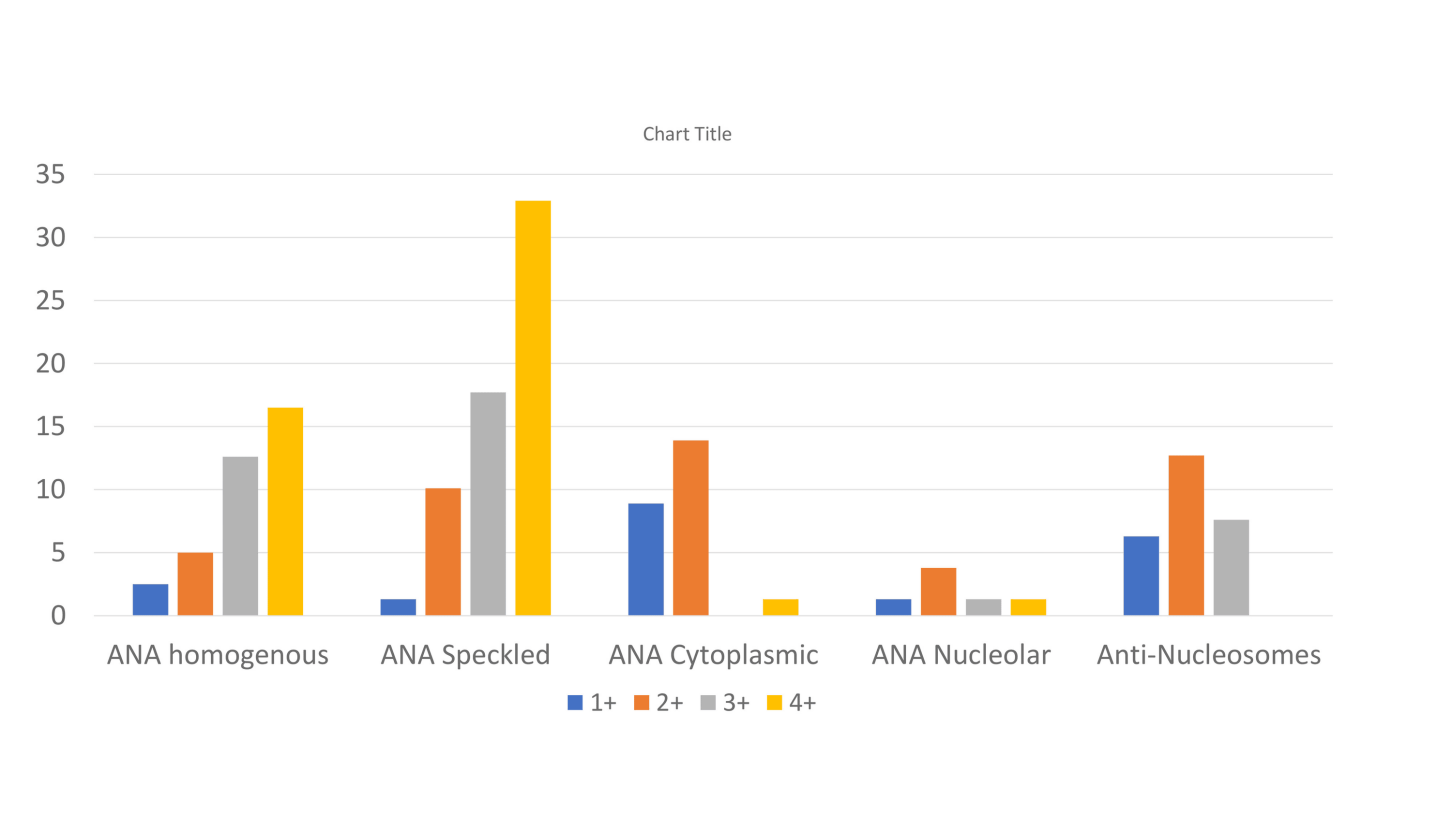
ANA screening profile of SLE patients in our study presented as percentage (%) with positivity level of titre ANA: Antinuclear antibodies, SLE: Systemic lupus erythematosus.

A mixed pattern was observed in 33 (41.8 %) patients. The speckled pattern was the most common among both female (44, 63.8%) and male (5, 50%) patients. In the pediatric population, speckled and homogenous patterns (5,50%) were both found to be commonly occurring ANA patterns. The most common antibody detected was anti-dsDNA (55, 69.6%) followed by anti-U1snRNP (47, 59.5%), anti-Sm (35, 44.3%), anti-Ro60 (35, 44.3%), and anti-Ro52 (31,39.2%). Figure 12 shows the percentage of patients with an antibody profile.

**Figure 10:**
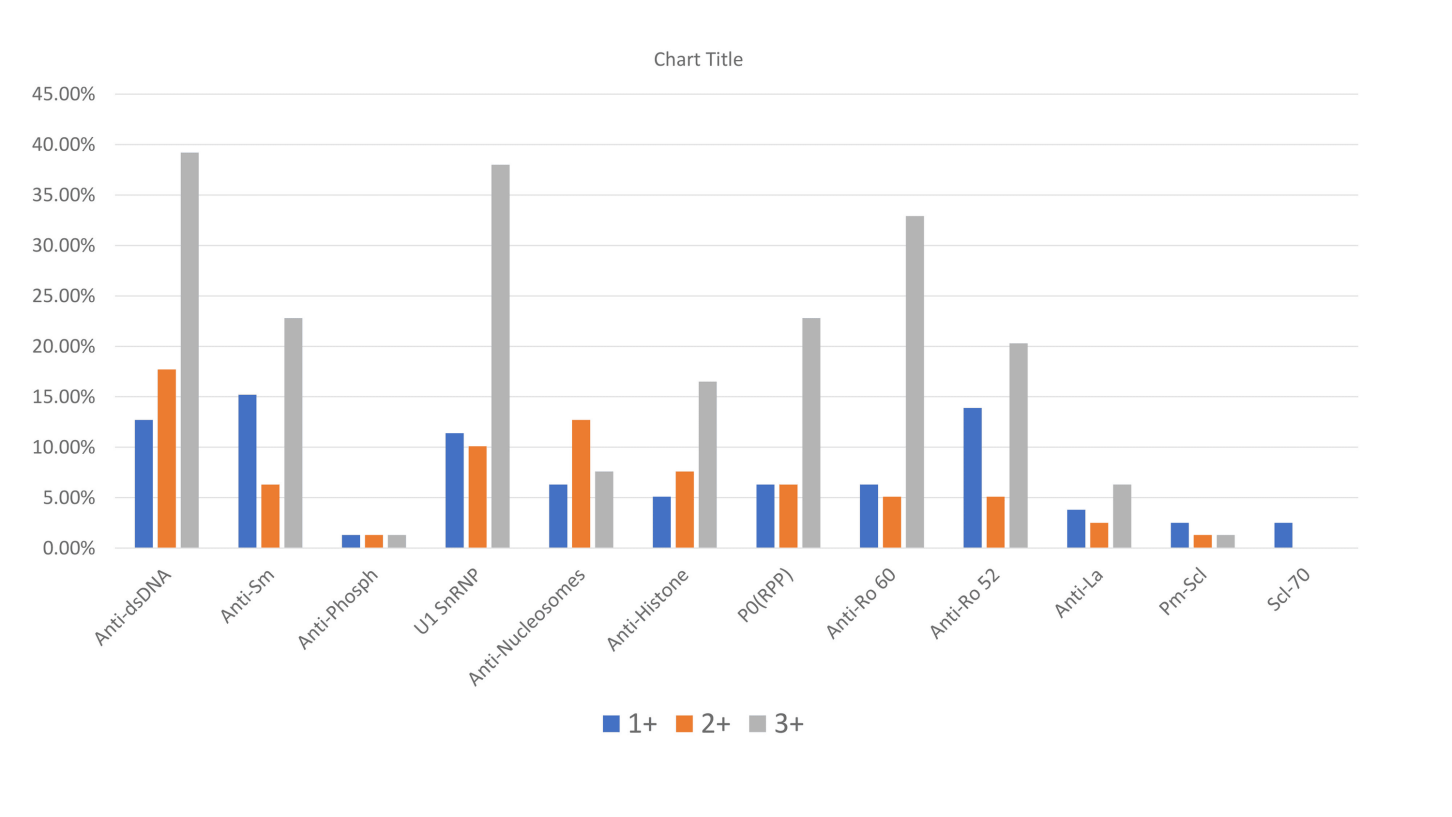
Data of patients with anti-nuclear antibody profile positivity in this study The data has been represented as a percentage (%).

In females, we came across anti-dsDNA (48, 69.6%), U1 snRNP (43, 62.3%), and anti-Ro60 (32, 46.4%) more frequently, while among males, anti-dsDNA (7, 70%), P0(RPP) (5, 50%), U1 snRNP (4, 40%) and anti-Sm (4, 40%) were the prevailing antibodies. In pediatric patients, anti-Sm (8, 80%) was the most common antibody, followed by anti-dsDNA (7, 70%), U1 snRNP (6, 60%), and P0(RPP) (6, 60%).

Association between various clinical manifestations and antibody profile

Anti-dsDNA was found to have a significant association with maculopapular rash (p-value = 0.012). It was also the frequently detected antibody in patients having malar rash, discoid rash, photosensitivity, oral ulcer, fever, and joint pain, but a statistically significant association could not be established. Anti-Sm antibody (25, 60.97%) was the most common antibody among LN patients with a significant association (p-value = 0.002). Anti-Sm antibody is known to be the most specific antibody associated with SLE, but our study is the first to find a significant association between LN and anti-Sm antibody among Indian patients. Speckled pattern was also the most common among patients having photosensitivity (39, 72.2%, p-value = 0.8), oral ulcer (38, 61.3%, p-value = 0.79), alopecia (41, 60.3%, p-value = 0.43), fever (39, 61.9%, p-value = 0.96), and joint pain (42, 62.7%, p-value = 0.77) respectively. Table 1 shows various clinical features, the commonly associated antibody, their percentages, and the respective p-values. 

**Table 1:** Various clinical features with commonly associated antibody (percentage N and p-value) The data is presented as N, percentage (%), and p-value (considered significant <0.05) using the Chi-square test. DLE: Discoid lupus erythematosus, ANA: Antinuclear antibody.

Clinical features	Commonly associated antibody	(N/%) of antibody present	p-value (considered significant <0.05)	Chi-Square Values
Malar rash	Anti-dsDNA	38, (69%)	0.55	0.78
Malar rash	U1SnRNP	35, (63.6 %)	0.25	1.29
Maculopapular	Speckled ANA pattern	28, (63.6%)	0.74	0.11
Maculopapular	Anti-dsDNA	27, (61.3%)	0.012	6.24
DLE	Anti-dsDNA	11, (78.6%)	0.32	0.35
DLE	Anti-Sm	9, (64.3%)	0.09	2.75
DLE	U1SnRNP	9, (64.3%)	0.69	0.16
Photosensitivity	Anti-dsDNA	36, (66.6%)	0.27	0.22
Photosensitivity	Speckled ANA pattern	34, (62.9%)	0.80	0.06
Oral ulcer	Anti-dsDNA	41, (66.1%)	0.16	0.48
Oral ulcer	Speckled ANA pattern	38, (61.3%)	0.79	0.06
Oral ulcer	U1SnRNP	37, (59.7%)	0.56	0.04
Alopecia	Anti-dsDNA	46, (67.6%)	0.28	0.17
Alopecia	U1SnRNP	41, (60.3%)	0.72	0.13
Alopecia	Speckled ANA pattern	41, (60.3%)	0.43	0.62
Fever	Anti-dsDNA	42, (66.6%)	0.21	0.03
Fever	Speckled ANA pattern	39, (61.9%)	0.96	0.002
Fever	U1SnRNP	37, (58.7%)	0.78	0.075
Joint pain	Anti-dsDNA	45, (67.2%)	0.22	0.72
Joint pain	Speckled ANA pattern	42, (62.7%)	0.77	0.30
Joint pain	U1SnRNP	39, (58.2%)	0.75	0.082
Lupus nephritis	Anti-Sm	25, (60.97%)	0.002	9.6
Lupus nephritis	U1SnRNP	26, (63.41%)	0.30	0.54
Lupus nephritis	Anti-dsDNA	27, (71.1%)	0.49	0.62

Treatment

Various treatment received by SLE patients include: oral prednisolone (0.5-1 mg/kg), hydroxychloroquine (HCQ-6.5 mg/kg), cyclophosphamide (1-1.5 mg/kg), azathioprine (1.5-2.5 mg/kg), mycophenolate mofetil (MMF, 1.5-3 g/day), methotrexate (0.3-0.5 mg/kg weekly), dexamethasone cyclophosphamide pulse (DCP, dexamethasone 100 mg intravenous (IV) for three consecutive days and cyclophosphamide 500 mg on day 2 in 500 ml of 5% dextrose solution monthly), dexamethasone azathioprine pulse (DAP, dexamethasone 100 mg IV for three consecutive days and oral azathioprine daily), dexamethasone mycophenolate mofetil pulse (dexamethasone 100 mg IV for three consecutive days and oral MMF daily) and injection rituximab (RTX, two loading doses of rituximab 1 gm, two weeks apart followed by 500 mg every six monthly maintenance dose) for treatment of different manifestations of LE. Those patients having contraindication to cyclophosphamide were given either azathioprine or MMF. Patients with high-grade proteinuria were administered rituximab based on their affordability and received other immunosuppressive agents like cyclophosphamide, azathioprine, or MMF in between periods.

All of our patients received oral steroids and HCQ. The patients of acute cutaneous LE responded well to oral prednisolone and were maintained on HCQ. Three patients (3.79%) of lupus profundus concomitantly having LN were treated with dexamethasone pulse with methotrexate. The patients with disseminated discoid lupus were treated with prednisolone and HCQ, to which they responded well. LN was further graded into four types based on the value of 24-hour urinary protein (Table 2).

**Table 2:** Grading of lupus nephritis according to the value of 24 hrs urinary protein The data has been represented as N, percentage (%).

Grading of Lupus nephritis	Value of 24 hr Urinary protein	Number of patients (N, %)
Grade 1	500-1000 mg	9, (21.95%)
Grade 2	1000-2000 mg	14, (34.14%)
Grade 3	2000-3500 mg	8, (19.5%)
Grade 4	>3500 mg	10, (24.39%)

For treating LN, DCP was given in 17 (41.5%) patients, and rituximab was administered in 13 (31.7%) patients. Three patients (1.23%) not responding to DCP were later administered rituximab. Patients with a contraindication to cyclophosphamide were treated with dexamethasone pulse and oral azathioprine (DAP) in six (14.6%) patients and dexamethasone pulse with MMF in five (12.2%) patients. Out of 17 patients who received DCP and oral cyclophosphamide, 14 (82.35%) patients responded well. Proteinuria resolved after two cycles of DCP in 12 patients (70.6%) and after three cycles of DCP in 14 patients (82.35%). Three patients (17.6%) showed persistent proteinuria with DCP and were shifted to injection RTX, to which they responded well. Six patients (14.6%) who received DAP showed resolution of proteinuria after a 3-pulse therapy. Similarly, five patients (12.2%) who received dexamethasone pulse with MMF showed improvement after a 3-pulse therapy. The majority of the patients received three to six cycles of dexamethasone pulse.

A total of 16 (39.02%) patients (three refractory cases to DCP) received an injection of rituximab for lupus nephritis, 15 (93.75%) of them showed excellent improvement, with only one patient (6.25%) showing persistent proteinuria after 12 months of therapy. Interestingly, in all these patients, there was no flare-up of skin lesions post-rituximab. However, four patients (100%) having bicytopenia (anemia and leukopenia) had relapse within one year of follow-up post RTX. For patients having hemolytic anemia, oral prednisolone was administered at 1 mg/kg, which was tapered gradually. Later, these patients were maintained on MMF. Table 3 illustrates the effect of rituximab on various systemic manifestations in SLE patients. 

**Table 3:** The details of patients taken rituximab with their systemic flare-up post-rituximab LN: Lupus nephritis.

S. No.	System involved	Baseline	Six months post-rituximab	12 months post-rituximab	18 months post-rituximab	24 months post-rituximab	36 months post-rituximab
Case 1	Skin	Malar rash, maculopapular rash, vasculitic lesions over palms and soles	Malar rash resolved with hyperpigmentation, vasculitic lesions resolved completely	No flare-up	No flare-up	No flare-up	No flare-up
	LN (24 hr urinary protein levels)	870	334	288	131	87	92
	Hematology	Pancytopenia	Anemia	resolved		No flare-up	No flare-up
Case 2	Skin	Maculopapular rash, bullous LE, diffuse erythema over palms & soles	Maculopapular rash resolved, bullous lesions resolved with hypopigmentation and scarring, erythema over palms and soles resolved completely	No flare-up	No flare-up	No flare-up	N/A
	LN (24 hr urinary protein levels)	2750	470	387	79	112	N/A
Case 3	Skin	Discoid lupus, malar rash	Discoid lesions resolved with hypopigmentation and atrophy, malar rash resolved with hyperpigmentation	No flare-up	No flare-up	N/A	N/A
	LN (24 hr urinary protein levels)	3325	227	190	115	N/A	N/A
Case 4	Skin	Diffuse erythema over photo-exposed sites, palms & soles, nail fold erythema and telangiectasia	Erythema resolved, nail fold erythema and telangiectasia resolved	No flare-up	No flare-up	No flare-up	N/A
	LN (24 hr urinary protein levels)	1112	449	361	329	478	N/A
Case 5	Skin	TEN like LE, vulval edema	TEN like LE resolved with hyperpigmentation, vulval edema resolved	No flare-up	No flare-up	No flare-up	No flare-up
	LN (24 hr urinary protein levels)	5380	1228	266	271	134	209
Case 6	Skin	Malar rash, patchy erythema over arms	Malar rash, patchy erythema resolved	No flare-up	No flare-up	N/A	N/A
	LN (24 hr urinary protein levels)	1565	211	285	422	N/A	N/A
Case 7	Skin	Maculopapular rash, discoid lupus	Discoid lesions resolved with hypopigmentation and atrophy, maculopapular rash resolved	No flare-up	No flare-up	N/A	N/A
	LN (24 hr urinary protein levels)	3270	765	267	81	N/A	N/A
Case 8	Skin	Malar rash, maculopapular rash, palmar erythema	Malar rash resolved, maculopapular rash resolved, palmar erythema resolved	No flare-up	No flare-up	No flare-up	No flare-up
	LN (24 hr urinary protein levels)	3980	1305	545	377	412	67
	Hematology	Pancytopenia	Anemia, thrombocytopenia	resolved	Anemia	resolved	No flare-up
Case 9	Skin	Discoid lupus, palmar erythema, nail fold erythema, telangiectasia	Discoid lesions resolved with hypopigmentation and atrophy, palmar erythema resolved, nail fold erythema, telangiectasia resolved	No flare-up	No flare-up	N/A	N/A
	LN (24 hr urinary protein levels)	2440	474	234	288	N/A	N/A
Case 10	Skin	Patchy erythema over photo-exposed areas, palms & soles	Erythema resolved	No flare-up	No flare-up	N/A	N/A
	LN (24 hr urinary protein levels)	3677	925	445	357	N/A	N/A
	Hematology	Anemia, leukopenia	leukopenia	resolved	No flare-up	N/A	N/A
Case 11	Skin	Malar rash, maculopapular rash	Resolved with hyperpigmentation	No flare-up	No flare-up	N/A	N/A
	LN (24 hr urinary protein levels)	2077	355	118	143	N/A	N/A
Case 12	Skin	Malar rash, discoid lupus, vasculitic lesions over palms and soles	Discoid lesions resolved with hypopigmentation and atrophy, malar rash resolved with hyperpigmentation, vasculitic lesions resolved completely	No flare-up	No flare-up	Patchy erythema over photo-exposed sites	No flare-up
	LN (24 hr urinary protein levels)	4558	1433	329	156	76	122
Case 13	Skin	Patchy erythema, bullous LE	Erythema resolved, bullous lesions resolved with atrophy and scarring	No flare-up	No flare-up	N/A	N/A
	LN (24 hr urinary protein levels)	2887	561	467	411	N/A	N/A
	Hematology	Anemia	resolved	No flare-up	Anemia	N/A	N/A
Case 14	Skin	TEN like LE, patchy erythema over palms and soles	TEN like LE resolved with hyperpigmentation, patchy erythema resolved	No flare-up	No flare-up	No flare-up	N/A
	LN (24 hr urinary protein levels)	8123	3223	1223	589	311	N/A
Case 15	Skin	Discoid lupus, lupus profundus	Lupus profundus induration decreased, discoid lesions resolved with hypopigmentation and atrophy,	No flare-up	No flare-up	N/A	N/A
	LN (24 hr urinary protein levels)	2334	256	322	139	N/A	N/A
Case 16	Skin	Malar rash, patchy erythema over forearms, palms & soles	Malar rash resolved, patchy erythema resolved	No flare-up	No flare-up	No flare-up	N/A
	LN (24 hr urinary protein levels)	1765	515	287	133	225	N/A

All the lupus hepatitis patients responded well to systemic corticosteroids. Patients with Raynaud’s phenomenon were given oral nifedipine (10-30 mg/day) with close blood pressure monitoring, depending on the severity of the disease. The patients with CNS lupus also responded well to oral prednisolone. The three cases of antiphospholipid antibodies (APLA) were initially managed with low molecular weight heparin (LMWH) and gradually shifted to warfarin with a target international normalized ratio (INR) of 2-3. Also, aspirin was subsequently started for long-term prophylaxis. The patients with HLH were managed by administering three cycles of DCP followed by maintenance on high-dose oral prednisolone 1 mg/kg for around six to eight weeks, a daily dose of cyclophosphamide (1 mg/kg), and HCQ (6.5 mg/kg). Out of three patients with vulval swelling, two (66.7%) had LN and received two cycles of DCP, and one (33.3%) had no renal involvement and was treated with prednisolone (1 mg/kg) and HCQ (6.5 mg/kg). All cases of vulval edema improved with treatment.

## Discussion

The present study aims to determine the clinical and immunological profile of SLE patients in eastern India and also compare the findings with various other studies from India. In our study, the majority of patients belonged to the age group of 18-40 years. This finding was similar to the study conducted by Santhanam et al. [13] and Kishor et al. [14], who reported that the 21-30 years age group was the most common age group affected. Female predominance was seen with 69 (87.3%) patients being female and a female-to-male ratio of 6.9:1. Talukdar et al. also reported female predominance with a female-to-male ratio of 28:1 in their study [15]. The mean age of the study population was 29.5 years. The eldest patient was 75 years old, while the youngest was 10 years old. Mean disease duration was 26.3 months. Ten patients (12.7%) belonged to the pediatric age group (<18 years).

Diffuse non-scarring alopecia (68, 86.1%), joint pain (67, 84.8%), fever (63, 79.7%), and oral ulcer (62, 78.5%) were the common clinical manifestations. These findings were slightly varying from studies conducted by Kishor et al. [14] and Santhanam et al. [13], who reported fever followed by arthritis and cutaneous manifestations to be the common clinical findings. Photosensitivity was reported among 54 (68.4%) patients in this study, which was similar to the findings of Zian et al., who also reported photosensitivity in 60% of patients [16]. Jagdish et al. reported a high percentage of CNS lupus (45%), unlike our study, where CNS involvement was present in only eight (10.1%) patients [17]. Rare clinical presentations seen were vasculitic ulcers (8, 10.1%), bullous lesions (5, 6.32%), urticarial vasculitis (3, 3.79%), and livedo reticularis (3, 3.79%) in the current study.

Diffuse non-scarring alopecia (9, 90%) was the most common clinical finding among pediatric patients, similar to adults (68, 86.1%) in our study. Vijay and Parveen in their study reported fever (81%) followed by malar rash (68%) and arthritis (56%) to be the common findings among the pediatric population [18]. Anemia (62, 78.4%), leukopenia (15,18.9%), and thrombocytopenia (11, 13.9%) were detected in this study. Talukdar et al. reported anemia in 61%, leukopenia in 7.8%, and thrombocytopenia in 8.5%, respectively [15]. LN (41, 51.9%) was a common finding among our patients. Similar rates of kidney involvement were also reported by Talukdar et al. [15] and Santhanam et al. [13] with 58% and 44%, respectively. On the other hand, a much lower rate of renal involvement was reported by Kosaraju et al. [19], Jagdish et al. [17], and Kishor et al. [14] with 20%, 21%, and 25% of patients, respectively.

In our study, seven (70%) of pediatric patients had renal involvement, which was comparable to the findings of Vijay and Parveen where 50% of patients were reported to have renal involvement [18]. Anemia, leukopenia, and thrombocytopenia were present among nine (90%), two (20%), and two (20%) of pediatric patients, which was comparable to the study findings of Vijay and Parveen, where 56%, 12.5%, and 31.2% of patients were involved, respectively [18].

ANA was found to be positive in 70 (88.9%) patients. Zian et al. [16] reported 88%, while Kosaraju et al. [19] reported 64.28%, and Jagdish et al. [17] reported 100% ANA positivity. Speckled pattern (49, 62.02%) was the most common, followed by homogenous (29, 36.7%), cytoplasmic (19, 24.1%), and nucleolar (6, 7.6%) patterns. The speckled pattern was the most common pattern among both female (44, 63.8%) and male (5, 50%) patients, unlike Kosaraju et al., who reported the homogenous pattern (55.5%) to be the most common [19]. 

The most common antibody detected was anti-dsDNA (55, 69.6%), followed by anti-U1snRNP (47, 59.5%), anti-Sm (35, 44.3%), anti-Ro 60 (35, 44.3%), and anti-Ro52 (31, 39.2%). In comparison, Talukdar et al. [15], Santhanam et al. [13], and Jagdish et al. [17] reported anti-dsDNA to be the most common antibody with 62.7%, 45% and 43.3% positivity, respectively. On the other hand, Kishor et al. reported anti-nucleosome (48%) followed by anti-U1snRNP (45%) to be the most prevalent antibody in their study [14].

The current treatment approach in SLE is based on the ‘treat-to-target’ principle and focuses on achieving a state of remission [20]. This approach includes the use of glucocorticoids and other immunosuppressive agents [21]. In this study, all the patients (100%) received oral steroids and HCQ. HCQ in the long term reduces the risk of organ damage and also delays disease flare-ups [22]. HCQ also diminishes the risk of renal flare-ups, end-stage renal disease (ESRD), and death [23]. On the other hand, glucocorticoids rapidly control disease activity and remain the cornerstone of therapy despite the various known adverse effects [24]. The recent recommendations suggest limited use of glucocorticoid therapy depending on the disease activity [25]. Other non-corticosteroid immunosuppressive agents used for the treatment of SLE include azathioprine, MMF, cyclophosphamide, methotrexate, and calcineurin inhibitors [26]. The choice of immunosuppressive agent depends on the activity of lupus and the severity of organ involvement [26]. For the treatment of LN, intravenous cyclophosphamide along with glucocorticoids is used for inducing remission in proliferative LN [27].

Among 41 (51.9%) patients of LN in our study, 17 patients (41.5%) received DCP followed by oral cyclophosphamide (1 mg/kg) and showed remission in 14 patients (82.4%) but two (11.7%) patients with grade 3 and one (5.9%) with grade 4 proteinuria were refractory to treatment after three cycles of DCP pulse and remission was achieved in these patients after two loading doses of rituximab. Dexamethasone pulse with MMF was used in five (12.2%) patients, and all of them achieved remission of LN after three pulses and were later maintained on oral MMF (2-3 gm/day). In comparison to cyclophosphamide, MMF may help achieve complete remission more frequently with a lesser side-effect profile, especially among the younger population, with the only disadvantage being the higher cost of therapy [28]. Similarly, DAP was used in six (14.6%) patients, and all the patients showed remission after three cycles and were maintained on oral azathioprine (2 mg/kg). According to a systematic meta-analysis, an increase in the relapse rate was noted in patients on azathioprine when compared to MMF for maintenance therapy [29]. Calcineurin inhibitors can also be used for treating LN, but were not used in our study [30]. Rituximab is another drug used for treating high-grade proteinuria and refractory cases of DCP. All the patients showed remission of LN after receiving two loading doses of rituximab following six months of therapy. Rituximab is an off-label indication for treating severe LN and lupus with neuropsychiatric symptoms [29].

One study participant with lupus profundus was treated with one cycle of dexamethasone pulse with methotrexate (15 mg/week). The disease activity was arrested and the patient was maintained on methotrexate therapy (15 mg/week). In two (66.7%) patients of LN associated with vulval edema, two cycles of DCP were given along with oral prednisolone and cyclophosphamide, and a complete resolution was seen. Another patient with vulval edema without LN was treated only with oral prednisolone (1 mg/kg) and HCQ, with which the patient responded well. Interestingly, we had two cases of HLH who were treated with three cycles of DCP along with oral prednisolone and HCQ. Secondary HLH is an uncommon but severe complication of SLE and is difficult to distinguish from active SLE flare-up, so clinicians should be well aware of this association [30]. All the patients with Raynaud’s phenomenon were well controlled on nifedipine. Another interesting case of lupus nephritis presented with urticarial vasculitis, for whom rituximab was administered along with oral steroids, but the urticarial vasculitis lesions continued to recur. Subsequently, the lesions were well controlled with tofacitinib 5 mg administered twice daily. Table 4 shows a comparison of similar studies from different parts of India with the present study.

**Table 4:** A comparison of similar studies from different parts of India with the present study The data has been represented as N, percentage (%). F:M: Female-to-male ratio, ANA: Antinuclear antibodies.

Author, year	F:M	Most common age group (years) and mean age	Most common clinical features	ANA positivity rate (%) and most common pattern	ANA Profile (Most common antibody)
Kosaraju et al. [19], 2010	15:1	21-30, 34.25	Arthritis, fever, malar rash	64.28%, Homogenous	Anti-dsDNA
Santhanam et al. [13], 2016	6:1	21-30	Fever, arthritis, cutaneous	100%	Anti-Sm, anti-Ro, anti-dsDNA
Kishor et al. [14], 2016	5.6:1	15-30, 29.8	Fever, arthritis, cutaneous	N/A	Anti-nucleosome, anti-U1 Sn RNP, anti-dsDNA
Talukdar et al. [15], 2020	28:1	21-30, 25.89	Mucocutaneous, hematological, renal	100%	Anti-dsDNA, anti-Ro60, anti-Ro52
Present study	6.9:1	18-40, 29.5	Diffuse non-scarring alopecia, joint pain, fever, oral ulcer	88.9%, Speckled	Anti-dsDNA, anti-U1 SnRNP, anti-Sm, anti-Ro60

Study limitations

As it was a hospital-based study with a relatively smaller sample size, extrapolating the findings of this study to the general population raises questions about the external validity of this study.

## Conclusions

Our study highlights the major clinical features and immunological profile of SLE patients in the eastern part of India. Female preponderance was seen with a female-to-male ratio of 6.9:1. Diffuse non-scarring alopecia was the most common clinical presentation, followed by joint pain, fever, and oral ulcer. ANA was detected in 70 (88.9%) patients, and the majority of them had a speckled pattern. Anti-dsDNA followed by anti-U1snRNP, Anti-Sm and Anti-Ro were frequently found antibodies. Anti-Sm antibody was found to be significantly associated with lupus nephritis, and rituximab was successfully used to treat high-grade LN cases in the majority of study participants. This is one of the first studies conducted in India where we determined the association between various clinical manifestations and antibody profiles.

## References

[1] Mathur R, Deo K, Raheja A (2022). Systemic lupus erythematosus in India: a clinico-serological correlation. Cureus.

[2] Malaviya AN, Singh RR, Singh YN, Kapoor SK, Kumar A (1993). Prevalence of systemic lupus erythematosus in India. Lupus.

[3] McCauliffe DP (2001). Cutaneous lupus erythematosus. Semin Cutan Med Surg.

[4] Gilliam JN, Sontheimer RD (1982). Skin manifestations of SLE. Clin Rheum Dis.

[5] (2024). Rook’s textbook of dermatology. https://academic.oup.com/bjd/article-abstract/191/5/854/7728241?redirectedFrom=fulltext.

[6] Kole AK, Ghosh A (2009). Cutaneous manifestations of systemic lupus erythematosus in a tertiary referral center. Indian J Dermatol.

[7] Cardinali C, Caproni M, Bernacchi E, Amato L, Fabbri P (2000). The spectrum of cutaneous manifestations in lupus erythematosus--the Italian experience. Lupus.

[8] (2012). Fitzpatrick’s dermatology in general medicine.

[9] Park DJ, Joo YB, Bang SY, Lee J, Lee HS, Bae SC (2022). Predictive factors for renal response in lupus nephritis: a single-center prospective cohort study. J Rheum Dis.

[10] Shrivastava A, Khanna D (2011). Autoantibodies in systemic lupus erythematosus: Revisited. Ind J Rheumatol.

[11] Basta F, Fasola F, Triantafyllias K, Schwarting A (2020). Systemic lupus erythematosus (SLE) therapy: the old and the new. Rheumatol Ther.

[12] Rajadhyaksha AG, Mehra S, Nadkar MY (2013). Biologics in SLE: the current status. J Assoc Physicians India.

[13] Santhanam S, Mani M, Tamilselvam TN, Rajeswari S (2016). Clinical and immunological profile of SLE patients: experience from a Chennai-based tertiary care centre (revisited). Int J Rheumatol Clin Immunol.

[14] Kishor N, Boloor R, Sukumar T (2016). A cross-sectional study of clinico-immunological profile of systemic lupus erythematosus patients in a tertiary care centre in Mangalore. Indian J Allergy Asthma Immunol.

[15] Talukdar D, Gogoi A, Doley D (2020). The clinical and immunological profiles of systemic lupus erythematosus patients from Assam, North-East India. Indian J Rheumatol.

[16] Zian Z, Maamar M, Aouni ME (2018). Immunological and clinical characteristics of systemic lupus erythematosus: a series from Morocco. Biomed Res Int.

[17] Jagdish GA, Va L, H KS (2022). Clinico-immunological profile of systemic lupus erythematosus: an observational study. J Assoc Physicians India.

[18] Vijay Y, Parveen B (2014). Clinical and immunological profile of systemic lupus erythematosus in a pediatric population in North India. Egypt Rheumatol Rehabil.

[19] Kosaraju K, Shenoy S, Suchithra U (2010). A cross-sectional hospital-based study of autoantibody profile and clinical manifestations of systemic lupus erythematosus in south Indian patients. Indian J Med Microbiol.

[20] van Vollenhoven R, Voskuyl A, Bertsias G (2017). A framework for remission in SLE: consensus findings from a large international task force on definitions of remission in SLE (DORIS). Ann Rheum Dis.

[21] Katarzyna PB, Wiktor S, Ewa D, Piotr L (2023). Current treatment of systemic lupus erythematosus: a clinician's perspective. Rheumatol Int.

[22] (1991). A randomized study of the effect of withdrawing hydroxychloroquine sulfate in systemic lupus erythematosus. N Engl J Med.

[23] Alarcón GS, Ugarte-Gil MF, Pons-Estel G, Vilá LM, Reveille JD, McGwin G Jr (2019). Remission and low disease activity state (LDAS) are protective of intermediate and long-term outcomes in SLE patients. Results from LUMINA (LXXVIII), a multiethnic, multicenter US cohort. Lupus.

[24] Little J, Parker B, Lunt M (2018). Glucocorticoid use and factors associated with variability in this use in the Systemic Lupus International Collaborating Clinics Inception Cohort. Rheumatology (Oxford).

[25] van Vollenhoven RF, Bertsias G, Doria A (2021). 2021 DORIS definition of remission in SLE: final recommendations from an international task force. Lupus Sci Med.

[26] Amissah-Arthur MB, Gordon C (2010). Contemporary treatment of systemic lupus erythematosus: an update for clinicians. Ther Adv Chronic Dis.

[27] Houssiau FA, Vasconcelos C, D'Cruz D (2002). Immunosuppressive therapy in lupus nephritis: the Euro-Lupus Nephritis Trial, a randomized trial of low-dose versus high-dose intravenous cyclophosphamide. Arthritis Rheum.

[28] Flanc R, Roberts M, Chadban S, Kerr P, Edworthy S, Atkins R (2000). Treatment for lupus nephritis. Cochrane Database of Systematic Reviews.

[29] Tunnicliffe DJ, Palmer SC, Henderson L (2018). Immunosuppressive treatment for proliferative lupus nephritis. Cochrane Database Syst Rev.

[30] Mok CC, Ho LY, Ying SK, Leung MC, To CH, Ng WL (2020). Long-term outcome of a randomised controlled trial comparing tacrolimus with mycophenolate mofetil as induction therapy for active lupus nephritis. Ann Rheum Dis.

